# *Letter to the Editor:* Importation of the First Bovine ST361 New Delhi Metallo-5 Positive *Escherichia coli* in Greece

**DOI:** 10.1089/mdr.2021.0243

**Published:** 2022-03-04

**Authors:** Katerina Tsilipounidaki, Zoi Athanasakopoulou, Charalambos Billinis, Vivi Miriagou, Efthymia Petinaki

**Affiliations:** ^1^ Department of Microbiology, Faculty of Medicine, University of Thessaly, Larissa, Greece.; ^2^ Department of Microbiology & Parasitology, Faculty of Veterinary Science, University of Thessaly, Karditsa, Greece.; ^3^ Laboratory of Bacteriology, Hellenic Pasteur Institute, Athens, Greece.

**Dear Editor:**

New Delhi Metallo (NDM)-β-lactamases producing bacteria have become a great threat to public health, due to their fast and wide geographical spread in recent years.^1^ These enzymes hydrolyze all β-lactams except aztreonam and are not inhibited by the novel β-lactamase inhibitors such as avibactam, relebactam, and vaborbactam. NDM was first reported in the literature in 2009 in a *Klebsiella pneumoniae* isolate and was referred to as NDM-1. To date, there have been 13 variants of NDM that have been identified: NDM-1 to NDB-14 (NDM-11 was not assigned to any unique variant).^1^

Several reports have demonstrated the dissemination of NDM among *Escherichia coli* isolated, not only from humans but also from animals (canine, horse, geese, chicken, *etc.*).^1^ Thessaly is a rural area in central Greece with 1,000,000 inhabitants, many of whom are professionally engaged in animal husbandry (sheep, bovines, and cattle). Given that in Greece the bovines are imported from countries where NDM-positive *E. coli* have been emerged, continuous surveillance for the detection of such strains is important. Here we describe the first bovine NDM-5 positive *E. coli* in central Greece.

From October to December 2020, a total of 213 nonduplicated fecal samples of clinically healthy bovines from 25 different farms in Thessaly were collected. For the collection of carbapenem-resistant bacteria, the samples were plated in the CHROMagar mSuperCARBA agar plates, prepared according to the manufacturer's instructions (≤72 hours of preparation) and were incubated for 24 hours at 35°C in air atmosphere. Each morphologically different colony grown on the plates was subcultured on MacConkey agar. Identification of the isolated bacteria and antimicrobial susceptibility testing were carried out using the automated Vitek-2 system (BioMérieux, Marcy l'Etoile, France), according to the manufacturer's instructions. Minimal inhibitory concentrations (MICs) to imipenem and meropenem were determined by MIC test strip (Liofilchem), whereas determination of colistin’ MIC was performed by broth microdilution method, according to European Committee on Antimicrobial Susceptibility Testing guidelines.^2^

Microorganisms that were resistant to any carbapenem were further tested for phenotypic carbapenem production using MIC test strips containing meropenem plus ethylenediaminetetraacetic acid (EDTA) and meropenem plus phenylboronic acid (Liofilchem). Isolates that had a ratio meropenem/meropenem plus EDTA ≥8 and/or meropenem/meropenem plus phenylboronic acid ≥8 were selected for molecular detection of carbapenemase encoding genes.

Bacterial DNA was extracted from overnight cultures of the selected microorganisms using the PureLink™ Genomic DNA Mini Kit (Invitrogen, Darmstadt, Germany), according to the manufacturer's instructions for Gram-negative bacteria. Detection of carbapenemase genes *bla*_VIM_, *bla*_NDM_, *bla*_KPC_, and *bla*_OXA-48_ was performed by PCR followed by sequencing analysis.

Surveillance cultures showed that only one microorganism, one *Escherichia coli* (B103), was resistant to carbapenems, with MICs of imipenem and meropenem 64 and 32 mg/L, respectively. The isolate was also resistant to all β-lactams except aztreonam, amikacin, gentamicin, tobramycin, ciprofloxacin, levofloxacin, moxifloxacin, trimethoprim/sulfamethoxazole, and chloramphenicol, whereas it was susceptible to tigecycline, fosfomycin, and colistin. Phenotypic detection of carbapenemase showed the presence of a metallo-β lactamase that was identified as NDM-5. *Escherichia coli* B103 was further analyzed by whole genome sequencing.

Genomic DNA was sequenced on a S5-Ion System platform. *In silico* multilocus sequence typing showed that the isolate belonged to ST361. The *Escherichia coli* B103 carried the following virulence genes *capU*, *gad*, *iss. sitA*, *terC*, and *traT*. Point mutations to *gyrA*, *parC*, and *parE* genes conferring resistance to fluoroquinolones were identified. In addition, the isolate-harbored genes conferring resistance to β-lactams [*bla*_NDM-5_ and *bla*_TEM-1_], aminoglycosides [*aadA1*, *aadA2*, *aph(3′)-1a*, *aph(3″)-Ib*, *aph(6)-id*, and *rmtB*], macrolides [*mph*(A)], phenicols [*cmlA1* and *floR*], sulfonamides [*sul1*, *sul2*, and *sul3*], tetracycline [*tet*(A) and *tet(*M)], and trimethoprim [*dfrA12*] were also detected. The *bla*_NDM-5_ gene was located on an IncFII plasmid, ∼100 kb in size, that exhibited high similarity to the p52148_NDM-5 plasmid found in an *E. coli* isolate from Czechia.^3^ The *bla*_NDM-5_ was part of a gene array comprising IS26—ΔISAba125—*bla*_NDM-5_—*ble*_MBL_—IS91 family transposase—*trpF*—*tat*—*sul1*—*qacEdelta1*—*aadA2* (Submission ID: SUB10011728, BioProject ID: PRJNA746426).

During the past 5 years (2015–2020) ∼1.9% (107 out 5660) of *E. coli* isolated from humans in Thessaly were resistant to carbapenems; among them 0.7% were positive for NDM-1 and belonged to various sequence types (STs) such as 744, 998, 410, 4380, 12, 683, and 46. So, the detection of the first bovine ST361 NDM-5 positive *E. coli* raised the question of its origin. Surveillance fecal cultures of the farm owner and the personnel gave negative results for carbapenem-resistant bacteria. Repetitive fecal cultures from the other animals' farm did not reveal any carbapenem-resistant microorganism. The animal was imported a month ago from Czechia and possibly it was already colonized.

It is known that ST361 NDM-5 positive *E. coli* have been detected in many countries such as South Korea, Japan, China, Switzerland, and Germany from humans, animals, and environment (rivers, sewage, and wastewater treatment plants).^3–5^ Screening of imported animals for carbapenem-resistant bacteria is a challenge because these micro-organisms colonize their gastrointestinal tract and can go undetectable.
